# Investigation of Biofilm Forming Ability in *Staphylococci* Causing Bovine Mastitis Using Phenotypic and Genotypic Assays

**DOI:** 10.1155/2013/378492

**Published:** 2013-11-02

**Authors:** Samah F. Darwish, Hanaa A. E. Asfour

**Affiliations:** ^1^Biotechnology Research Unit, Animal Reproduction Research Institute (ARRI), Giza, Egypt; ^2^Department of Mastitis and Neonatal Diseases, Animal Reproduction Research Institute (ARRI), Giza, Egypt

## Abstract

A total of 40 *S. aureus* and 68 coagulase negative *Staphylococcus* (CNS) isolates from bovine subclinical mastitis were investigated for their ability to form biofilm as one of the most important virulence factors.Using Congo Red Agar (CRA) method, 32.5%, 35%, and 32.5% of *S. aureus* strains were strong, intermediate, and negative biofilm producers, while in CNS the percentages were 29.5%, 42.6%, and 27.9%, respectively. By microtiter plate (MTP) method, 52.5%, 27.5%, and 20% of *S. aureus* isolates were strong, moderate, and weak biofilm producers, while in CNS the percentages were 44%, 30.9%, and 19.2%, respectively. Indian ink staining was used to detect the EPS layer of biofilm producers. All isolates were screened for presence of biofilm related genes, *eno, icaA, icaD,* and *bap.* In *S. aureus* isolates, the positive rates of *eno, icaA, icaD,* and *bap* genes were 75%, 15%, 62.5%, and 2.5% while in CNS were 92.6%, 5.9%, 47.1%, and 4.4%, respectively. The *eno* gene had the highest rate while the *bap* gene had the lowest rate. Presence of *icaA* and *icaD* genes was not always correlated with biofilm production. This study demonstrated high prevalence of *Staphylococcus* biofilm producers among bovine mastitis in Egypt. Therefore, attention must be paid toward implementation of new ways for effective treatment of such infections.

## 1. Introduction

Bovine mastitis caused by *S. aureus* and coagulase negative *Staphylococci* (CNS) remains a substantial problem for milk producers worldwide. The pathogenesis of *Staphylococcus* mastitis is attributed to a combination of extracellular factors and properties such as adherence and biofilm formation [1, 2].

Biofilm is an exopolysaccharide, a slime matrix around multiple layers of cells. The ability of *Staphylococci* to form biofilms is one of the virulence factors that facilitate the adherence and colonization of *Staphylococci* on the mammary gland epithelium, also contributing to the evasion of the immunological defences and to the difficulty of pathogen eradication, leading to recurrent or persistent infections [3–8].

*Staphylococcus* biofilm formation mechanisms are complex and include the participation of many kinds of proteins, and so many genes are involved. It is considered to be a two-step process. Firstly, the bacteria adhere to a surface mediated by a capsular antigen, namely, capsular polysaccharide/adhesin (PS/A). Then, the bacteria multiply to form a multilayered biofilm, which was associated with production of polysaccharide intercellular adhesin (PIA). The intercellular adhesion (*ica*) locus consisting of the genes *icaADB* and *C *encodes the proteins mediating the synthesis of PIA and PS/A in staphylococcal species [1, 9].

Monitoring the biofilm forming ability of *Staphylococci* causing mastitis and the genes involved in it may provide new ideas or strategy for the prevention or the effective treatment of bovine mastitis. According to our knowledge, in Egypt, researches on biofilm only began recently and mainly focused on *Staphylococcus* clinical isolates from human sources [10, 11]. So, the aim of the present study was to investigate the biofilm forming ability of *Staphylococcus* strains causing bovine mastitis using different phenotypic methods. Moreover, the detection of some biofilm related genes in these strains and their involvement with biofilm will be evaluated.

## 2. Material and Methods

### 2.1. Bacterial Isolates

The investigation was carried out on 108 *Staphylococcus* isolates recovered from bovine subclinical mastitic milk. The isolates were identified by their cultural characteristics on blood agar, mannitol salt agar, and Baird Parker agar media, microscopic appearance in Gram stained preparations, positive catalase reaction, hemolysin, and coagulase test (tube method) with rabbit plasma and biochemical analysis according to Quinn et al. [12].

### 2.2. Biofilm Formation Assays

#### 2.2.1. Phenotypic Analysis

*(1)  Detection of Slime Production by Congo Red Agar Method.* Slime production was evaluated by cultivation of all *Staphylococcus* isolates on Congo Red Agar (CRA) plates as described by Mathur et al. [13] (2006). Briefly, CRA plates were prepared using Tryptic Soy agar containing 0.08% Congo red (Sigma). The inoculated CRA plates were incubated at 37°C in aerobic conditions for 24 h, followed by storage at room temperature for 48 h [4]. Isolates were interpreted according to their colony phenotypes [14]. Black colonies with dry consistency and rough surface and edges were considered a positive indication of slime production, while both black colonies with smooth, round, and shiny surface and red colonies of dry consistency and rough edges and surface were considered as intermediate slime producers. Red colonies with smooth, round, and shiny surface were indicative of negative slime production.

*(2)  Detection of Biofilm by Microtiter Plate (MTP) Method*. All *Staphylococcus* isolates were grown overnight at 37°C as pure cultures on blood agar. Groups of three single colonies were inoculated in 3 mL Tryptone Soya broth. Suspensions were incubated for 24 h at 37°C and then diluted at 1 : 40 in a fresh TSB (2–7 × 10^7^ cfu/mL) using 0.5 MacFarland standard tube. This dilution was used as the inoculum in the microtiter plate test. Microtiter plate test was performed according to Dubravka et al. [14] and Stepanović et al. [15]. For each *Staphylococcus* isolate, 200 *μ*L aliquots of prepared suspension were inoculated into four wells of the 96-well tissue culture plates (Nunclon Delta, Nunc, Roskilde, Denmark). Each culture plate included a negative control, four wells with TSB. The plates were incubated at 37°C for 24 h. Afterwards, content of each well was removed by aspiration and the wells were rinsed three times with 250 *μ*L sterile physiological saline. The plates were dried in inverted position. The attached bacteria were fixed for 15 minutes at room temperature by adding 200 *μ*L volumes of methanol into each well. The plates were stained with 160 *μ*L aqueous solution of crystal violet 0.5% (Crystal Violet, Fluka) for 15 minutes at room temperature. Following staining, the plates were rinsed under running water until there was no visible trace of stain. The stain bound to bacteria was dissolved by adding 160 *μ*L of 95% ethanol. The optical density (OD) of each well was measured using a microplate ELISA reader (*E*_max⁡_, USA) at 570 nm. Cut-off OD (ODc) is defined as three standard deviations above the mean OD of the negative control. Strains were interpreted as follows:

1. nonbiofilm producers (OD ≤ ODc);
2. weak biofilm producers (ODc < OD ≤ 2 × ODc);
3. moderate biofilm producers (2 × ODc < OD ≤ 4 × ODc);
4. Strong biofilm producers (4 × ODc < OD).

*(3) Detection of Exopolysaccharide Production (EPS)*. Possible EPS-producing isolates were identified via their mucoid or ropy appearance. EPS production was assessed by Indian ink (Art. Material S.A., China) staining of wet mount preparation of each strain after overnight growth on CRA and examined under light microscope [16, 17]. Appearance of a transparent halo zone around the *Staphylococcus* cells denoted the existence of an organized EPS structure.

### 2.3. Genotypic Analysis

Four biofilm related genes were analyzed by simplex PCR assays to detect the presence of *icaA* (intercellular adhesion gene *A*), *icaD *(intercellular adhesion gene *D*), *bap* (encoding biofilm associated protein), and *eno* (encoding laminin binding protein) in all *Staphylococcus* isolates. First, crude DNA of the tested strains was extracted using a rapid boiling procedure according to Reischl et al. [18]. Two to 5 loops of *Staphylococcus* isolates taken from the brain heart infusion agar plate were collected and suspended in 200 *μ*L of lysis buffer comprised of 1% Triton X-100, 0.5% Tween 20, 10 mM Tris-HCl (pH 8.0), and 1 mM EDTA. After boiling for 10 min, the suspension was centrifuged for 2 min. to sediment bacterial debris. The supernatant was aspirated, from which 5 *μ*L was used directly for PCR amplification. Because of nonavailability of positive controls for the four genes and to exclude any false negative results, the DNA of all isolates was firstly examined by amplification of 16S rRNA gene using *Staphylococcus* genus specific primers. The names of target genes, nucleotide sequences of primers, references, annealing temperature, and amplicon sizes are shown in Table 1. The amplification cycles were carried out in a PT-100 Thermocycler (MJ Research, USA). With the exception of specific annealing temperatures mentioned in Table 1, the reaction condition was optimized to be 94°C for 4 min. as initial denaturation, followed by 40 cycles of 94°C for 60 seconds, annealing for 60 seconds and 72°C for 60 seconds. A final extension step at 72°C for 10 min. was followed. PCR condition was optimized using a total volume of 25 *μ*L reaction mixtures which contained 5 *μ*L of DNA as template, 25 pmol of each primer, and 1X of PCR master mix (Dream Taq Green PCR Master Mix, Fermentas Life Science). PCR products were analyzed by electrophoresis in 1.5% agarose gel in 0.5X TBE buffer containing ethidium bromide and visualized under a UV transilluminator.

### 2.4. Statistical Analysis

Data were presented as count and percentage. Sensitivity and specificity of CRA method were calculated according to Ilstrup, [22] using 2 × 2 table and MTP method as gold standard. The kappa coefficient test was also used to determine the agreement between the results obtained by the CRA method.

## 3. Results

### 3.1. Bacterial Isolation and Identification

The 108 *Staphylococcus* isolates were identified to be 40 *S. aureus* and 68 CNS. 

### 3.2. Biofilm Formation

#### 3.2.1. Slime Production on Congo Red Agar (CRA)

**Figure 1:**
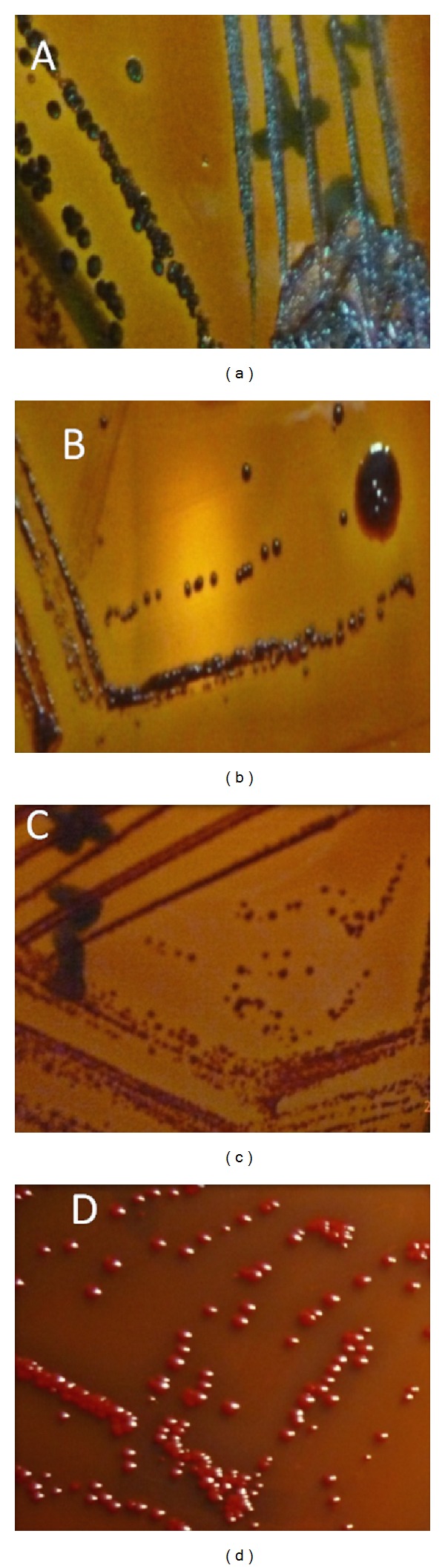
Screening of biofilm (slime) producer *Staphylococci* using Congo red agar plate method ((a) dry black colonies; (b) smooth black colonies; (c) dry red colonies; (d) smooth red colonies).

The results of biofilm production by *S. aureus* and CNS using CRA method are demonstrated in Table 2. About 70.4% of the isolates were positive for biofilm production with varied degree. Out of 40 *S. aureus* strains, 32.5%, 35%, and 32.5% were strong, intermediate, and negative biofilm producers, respectively. Among the 68 CNS strains, 29.5%, 42.6%, and 27.9% were strong, intermediate, and negative biofilm producers, respectively. Totally, from the 108 *Staphylococcus* isolates, 33 (30.6%), 27 (25%), 16 (14.8%), and 32 (29.6%) were dry black, smooth black, dry red, and smooth red, respectively. Morphology of all types of colonies is showed in Figure 1. 

#### 3.2.2. Biofilm Formation by MTP Method

**Figure 2:**
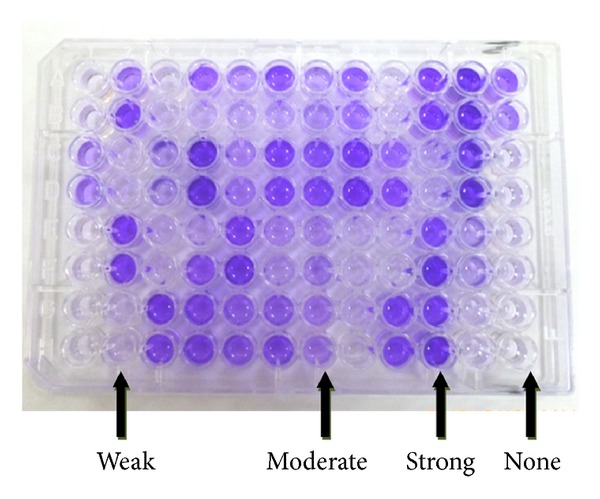
2 Microtiter plate method showing none, strong, moderate, and weak biofilm producers differentiated by crystal violet stain in 96-well tissue culture plate.

As shown in Figure 2 and Table 3, approximately 96.3% of the isolates were biofilm producers with MTP method, although production level varied. The biofilm production of *S. aureus* isolates (100%) was slightly higher than that of CNS isolates (94.1%). In the biofilm positive *S. aureus* strains, 52.5% of isolates were strong producers, while 27.5% and 20% were moderate and weak, respectively. Out of the biofilm positive CNS isolates, 44%, 30.9%, and 19.2% were strong, moderate, and weak, respectively.

Comparison of results obtained by CRA method versus that of MTP method is declared in Table 4. Out of 32 biofilm negative *Staphylococcus* isolates by CRA method, 28 isolates were positive by MTP method but with different degrees of production (4 strong, 8 moderate, 16 weak) and only 4 CNS isolates were true negative by both methods. CRA method showed little correlation with MTP assay where only 76 (70.4%) of the isolates were positive by both methods, kappa coefficient 0.167 slight agreement. The sensitivity and specificity of CRA method versus MTP method as gold standard were calculated to be 73.1% and 100%, respectively.

#### 3.2.3. Detection of EPS Production

**Figure 3:**
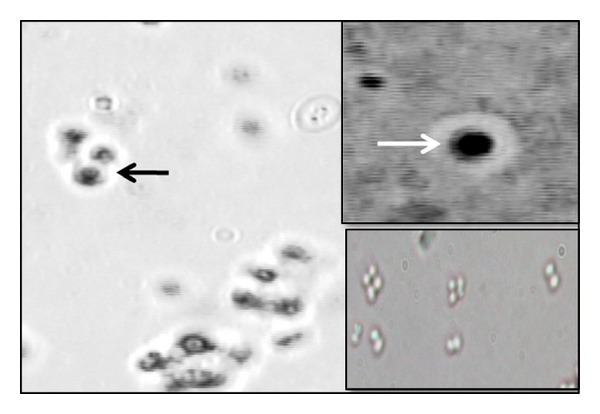
Morphology of some of *Staphylococcus* isolates as observed after staining with Indian ink. The arrows pointed to capsular-like EPS (light microscopy ×100).

All the 108 *Staphylococcus* isolates except the 4 biofilm negative CNS showed a distinct halo transparent zone surrounding the grapes linked cells similar to a capsule representing EPS layer (Figure 3).

### 3.3. Genotypic Analysis of Biofilm Related Genes

**Figure 4:**
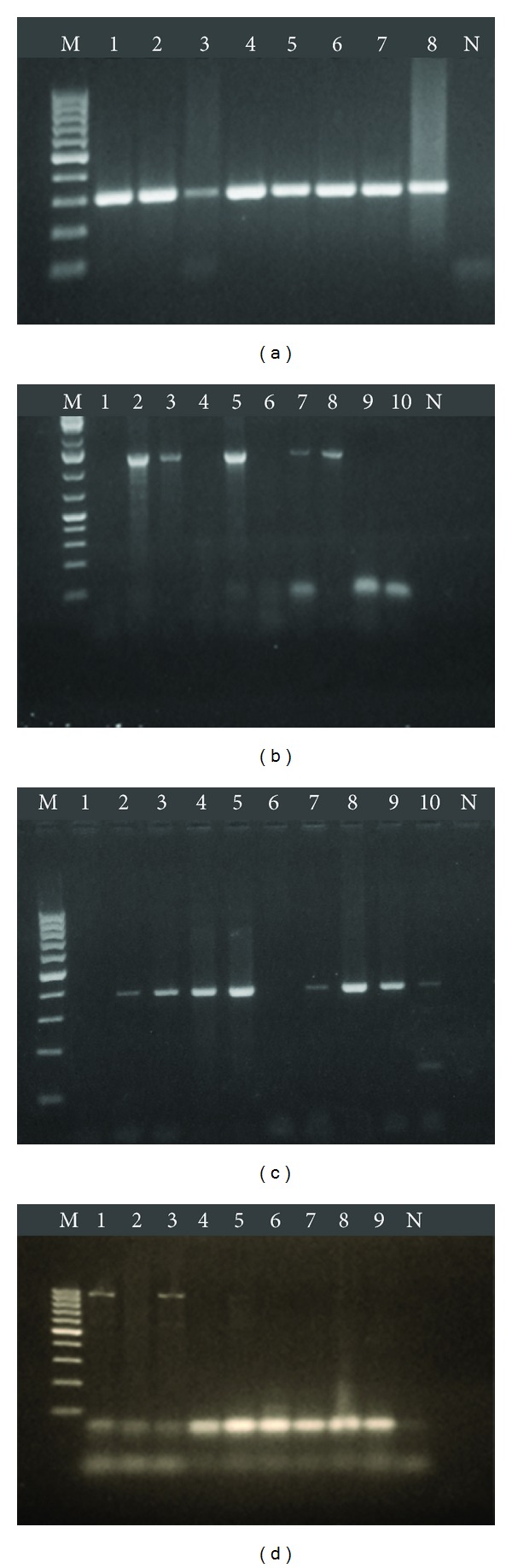
Agarose gel electrophoresis of PCR products stained with ethidium bromide. (a) *eno* gene (302 bp), M: 100 bp ladder DNA marker, 1–8: positive samples, N: negative control. (b) *icaA* gene (1315 bp), M: 1 kb plus DNA marker, 2, 3, 5, 7, 8: positive samples, 1, 4, 6, 9, 10: negative samples, N: negative control. (c) *icaD* gene (381 bp), M: 100 bp ladder DNA marker, 2–5, 7–10: positive samples, 1, 6: negative samples, N: negative control. (d) *bap *gene (971 bp), M: 100 bp ladder DNA marker, 1, 3: positive samples, 2, 4–9: negative samples, N: negative control.

**Figure 5:**
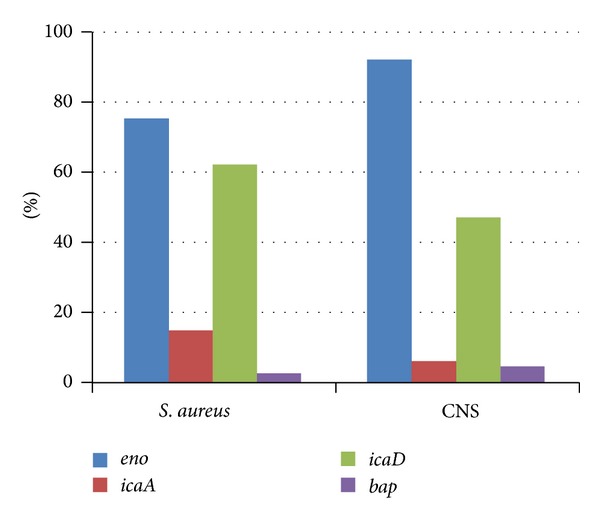
Prevalence of biofilm related genes in *S. aureus* and CNS isolated from bovine mastitis milk.

All the four biofilm related genes, *eno, icaA, icaD,* and *bap,* could be detected in both *S. aureus* and CNS isolates under study (Figure 4) but with varied prevalence (Figure 5). In *S. aureus* isolates, the positive rates of *eno, icaA, icaD,* and *bap* genes were 75%, 15%, 62.5%, and 2.5%, respectively (Table 5 and Figure 5). The prevalence of *eno, icaA, IcaD,* and *bap* genes in CNS isolates were 92.6%, 5.9%, 47.1%, and 4.4%, respectively (Figure 5 and Table 5). The prevalence of biofilm related genes varied between *S. aureus* and CNS. The results indicated that the *eno* gene had the highest rate in CNS (92.6%) and *S. aureus* (75%) followed by *icaD* gene which was detected in 62.5% of *S. aureus* and 47.1% of CNS. The prevalence of *icaA* gene was low versus that of *icaD* gene. The *bap* gene was detected in only 4 isolates, one *S. aureus* isolate (2.5%) and 3 CNS isolates (4.4%).

## 4. Discussion

Biofilms are related to pathogenicity, and it has been proposed that *Staphylococcus  *biofilms are major causes of recurrent and chronic mastitis in dairy cattle [23]. Numerous authors stated that bacteria in a biofilm are more resistant to antibiotics than in their planktonic form [24, 25].

In this study, slime production was examined qualitatively, depending on colony morphology of 108 bovine mastitis isolates of genus *Staphylococci* (*S. aureus* and CNS) produced on Congo red agar. Some differences between researches were apparent with respect to interpretation of CRA test results. In that respect, both bright black colonies [26] and black colonies [8, 27] were considered as a positive result. However, Cucarella et al. [5] described the dry crystalline surface (rough colony morphology) as a positive result, disregarding the color (black or pink). Such discrepancy when interpreting the results may possibly be due to the fact that the test itself was not originally designed for investigating *S. aureus *isolates as reported by Freeman et al. [28]. In this investigation, according to Dubravka et al. [14] isolates that formed black/rough colonies were recorded as strong slime producing, whereas isolates forming red/smooth colonies were described as nonslime producers. The smooth black and dry red colonies were considered as indeterminate result. As displayed in Table 2, slime production was detected in 33 (30.6%) isolates that produced characteristic black colonies of dry crystalline consistency while 32 isolates (29.6%) produced smooth red colonies. Also, 27 isolates (25%) produced black colonies with smooth shiny surfaces were interpreted as indeterminate result. Furthermore, 16 isolates (14.8%) produced red colonies with dry rough consistency were described as indeterminate result. Totally, 76 isolates (70.4%) were found to be slime producers with variable degrees.

MTP method was reported to have high specificity, sensitivity, and positive predictive value [13]. As shown in Table 3, using MTP method, 96.3% of the isolates were biofilm producers with variable production levels. This high prevalence agreed with that reported by Seo et al. [29] who noted that approximately 80% of their isolates produced slime with the MTP method but with variable degrees of production. On the contrary, lower prevalence rates of biofilm producers among *S. aureus* and *S. epidermidis* bovine mastitis isolates were also reported to be 29.41% [30] and 37.5% [8]. Comparison of results obtained by CRA method versus that of MTP method was declared in Table 4. Out of 32 biofilm negative *Staphylococcus* isolates (13 *S. aureus* and 19 CNS) by CRA method, 28 isolates were positive by MTP method but with different degrees of production (4 strong, 8 moderate, and 16 weak) while only 4 CNS isolates were negative by both methods. Congo red agar (CRA) method showed little correlation with MTP method where only 76 (70.4%) of the isolates were positive by both methods with kappa coefficient 0.167 (slight agreement). The sensitivity and specificity of CRA method versus MTP method as gold standard were calculated to be 73.1% and 100%, respectively. Although CRA method was easy to perform and less time consuming, however, our findings confirm what was reported by Mathur et al. [13] that CRA method cannot be recommended for detection of biofilm formation by *Staphylococci* alone. 

Difference between results of CRA and MTP methods can be attributed to the fact that phenotypic expression of biofilm formation is highly sensitive to *in vitro* conditions and hence can be detected variably by different methods. Also, both tests measure the same phenomenon but in different ways. CRA has been used as indirect indicator of polysaccharide production [16, 31].

Additionally, Indian ink was used to stain wet preparations of *Staphylococcus* isolates; all the 108 isolates except 4 biofilm negative CNS showed distinct halo transparent zones denoting to EPS layer surrounding the cells (Figure 3). The size of the hallo zones enlarged with the ability of the isolates to produce biofilms. This result proposed the use of Indian ink staining as a rapid screening test for biofilm formation when other methods are not feasible.

As combination of phenotypic and genotypic methods would be recommended for identifying biofilm producing strains, the detection of 4 biofilm related genes and their incidence in *Staphylococcus* isolates were investigated using simplex PCR reactions (Figures 4 and 5). Because of the absence of a reference positive control for each of the studied genes, the DNA of all isolates was first examined by *Staphylococcus* genus specific primers to monitor the quality of the DNA for amplification and to exclude any false negative results. All the 108 *Staphylococcus* isolates successfully amplified the specific 228 bp of the *16S rRNA* gene of genus *Staphylococcus*. This amplification confirmed all the isolates to be *Staphylococci* and ensuring the suitability of the DNA for amplification.

The intercellular adhesion (*ica*) locus, consisting of the genes *icaADBC*, has been reported to have a potential role as a virulence factor in the pathogenesis of mastitis in ruminants [4]. Among the *ica* genes, *icaA* and *icaD* have been reported to play a significant role in biofilm formation in *S. aureus* and *S. epidermidis* [32]. The prevalence rates of *icaA* and *icaD* genes were 15% and 62.5% in *S. aureus* isolates and 5.9% and 47.1% in CNS isolates, respectively (Figure 5). Although the majority of researches reported the *icaA* and *icaD* to be nearly similar in incidence [2, 4, 30], our results agreed with**  **Ciftci*  *et al. [33] who found that 16 and 38 out of 59 strains were positive for *icaA *and *icaD *genes, respectively. This difference in the prevalence rates can be attributed to variation in DNA sequences which may lead to failed amplification of the gene in some isolates leading to false negative results [34]. This was previously reported by Simojoki et al. [35] who excluded *icaA* and *icaD *results from their work because their primers, although were able to detect the genes in *S. aureus* isolates, were not able to detect the genes in known *icaA* and *icaD* positive *S. epidermidis*. 

Detection of *icaA* and *icaD* was not well correlated with biofilm production on MTP methods (Table 5). Presence of *icaA* or *icaD* negative biofilm positive isolates can be accounted for by an *ica* gene independent control of slime production/adhesion mechanism [36]. On the contrary, inability of *Staphylococcus* isolates that were positive for *icaA* and/or *icaD* genes to produce biofilm *in vitro* can be due to point mutation in the locus and/or any other yet unidentified factors that negatively regulate polysaccharide intercellular adhesion synthesis or influence biofilm formation [1]. Also, some experimental evidence supports the development of new clones referred as biofilm negative with both *icaA* and *icaD* genes positive [37]. Additionally, lacking of *icaA* mRNA or *icaD* mRNA or both was reported to explain absence of phenotypic expression of biofilm [36]. Despite discrepancy between results of *ica* locus and biofilm phenotypic expression, Vasudevan et al. [4] argued that a better methodology for biofilm detection is to screen strains for *ica* genes in addition to CRA or MTP methods not to miss the genotypically positive phenotypically negative strains. On the contrary, Ciftci et al. [33] reported that PCR targeting *icaA *and *icaD *genes may be not sufficient to detect slime production, and further studies targeting other genes should be conducted for accurate evaluation of slime production characters of *S. aureus *strains.

Among the studied genes, *eno* encoding the laminin-binding protein was the most commonly detected gene in *Staphylococcus* mastitis isolates under study. The prevalence rates of the *eno* gene were 75% and 92.6% in *S. aureus* and CNS, respectively (Table 5 and Figure 5). This high prevalence agreed with that of Simojoki et al. [35] who reported the same gene to be the most commonly detected in CNS isolates from mastitis in a percentage of 75%. Also, this gene was detected with the highest rate in airborne *Staphylococci* (83%) when compared with animal isolates (56%) as reported by Seo*  *et al. [29].

The *bap* gene encoding the biofilm associated protein was the least detected gene where it was only detected in one *S. aureus* (2.5%) isolate and 3 (4.4%) CNS isolates (Table 5 and Figure 5). This very low prevalence was previously reported by Cucarella et al. [5] who detected the *bap* gene in 5% of *S. aureus* obtained from bovine subclinical mastitis. This gene is also detected in other *Staphylococcus* species, including *S. epidermidis*, *S. chromogenes*, *S. simulans,* and *S. hyicus* [34]. Other authors did not detect the *bap* gene in their *Staphylococcus* isolates [29, 35, 38].

Antimicrobial therapy of mastitis is based on results of susceptibility tests *in vitro*; therefore, new tests must be adopted to select the antibiotic of choice for treatment of biofilm positive strains where ordinary test selects antibiotics that are effective only in inhibiting planktonic bacterial population, whereas bacteria in biofilm resist and survive the treatment and provide materials for further growth [39]. 

## 5. Conclusions

Findings of the present study demonstrated the great ability of both *S. aureus* and CNS bovine mastitis isolates to form biofilms with different degrees of production using different methods. This must be considered as an alarming situation, and so attention must be paid toward implementation of new ways for effective prophylaxis, control, and treatment of such infections in the dairy farms.

## Figures and Tables

**Table 1:** Primers used in the study, their nucleotide sequence, annealing temperatures, amplicon sizes, and their references.

Gene targeted	Primer sequence 5′-3′ (references)	Annealing temperature °C	Amplicon size bp
*16S rRNA *	GTA GGT GGC AAG CGTTAT CC CGC ACA TCA GCG TCA G Monday and Bohach [19]	55	228

*icaA *	CCTAACTAACGAAAGGTAG AAGATATAGCGATAAGTGC Vasudevan et al. [4]	49	1315

*icaD *	AAACGTAAGAGAGGTGG GGCAATATGATCAAGATAC Vasudevan et al. [4]	49	381

*Eno *	ACGTGCAGCAGCTGACT CAACAGCATYCTTCAGTACCTTC Tristan et al. [20]	55	302

*Bap *	CCCTATATCGAAGGTGTAGAATTG GCTGTTGAAGTTAATACTGTACCTGC Cucarella et al. [21]	60	971

**Table 2:** Biofilm formation in *S. aureus* and CNS isolates according to CRA method.

Species (no.)	No. (%) of isolates				
	Positive result	Intermediate result		Negative result	Total positive
	Dry black	Smooth black	Dry red	Smooth red	
*S. aureus* (40)	13 (32.5)	8 (20)	6 (15)	13 (32.5)	27 (67.5)
CNS (68)	20 (29.5)	19 (27.9)	10 (14.7)	19 (27.9)	49 (72.1)

Total (108)	33 (30.6)	27 (25)	16 (14.8)	32 (29.6)	76 (70.4)

**Table 3:** Biofilm formation in *S. aureus* and CNS isolates according to MTP method.

Species (no.)	No. (%) of isolates				
	Strong	Moderate	Weak	Non	Total positive
*S. aureus* (40)	21 (52.5)	11 (27.5)	8 (20)	0 (0)	40 (100)
CNS (68)	30 (44)	21 (30.9)	13 (19.2)	4 (5.9)	64 (94.1)

Total (108)	51 (47.2)	32 (29.7)	21 (19.4)	4 (3.7)	104 (96.3)

**Table 4:** CRA method versus MTP method for detection of biofilm formation by *S. aureus* and CNS isolates.

Species	CRA	No.	MTP method			
			Strong	Moderate	Weak	Non
*S. aureus *	Dry black	13	12	1	0	0
	Smooth black	8	5	2	1	0
	Dry red	6	1	5	0	0
	Smooth red	13	3	3	7	0

	Total	40	21	11	8	0

CNS	Dry black	20	17	3	0	0
	Smooth black	19	8	9	2	0
	Dry red	10	4	4	2	0
	Smooth red	19	1	5	9	4

	Total	68	30	21	13	4

**Table 5:** Association between results of MTP method and positive amplification of biofilm related genes in *S. aureus* and CNS isolates.

Species	MTP	No.	+*eno *		*+icaA *		+*icaD *		+*bap *	
			No.	%	No.	%	No.	%	No.	%
*S. aureus *	Strong	21	17	81	5	24	13	62	0	0
	Moderate	11	9	82	1	9	9	82	0	0
	Weak	8	4	50	0	0	3	37.5	1	12.5
	None	0	0	0	0	0	0	0	0	0

	Total	40	30	75	6	15	25	62.5	1	2.5

CNS	Strong	30	30	100	3	10	18	60	1	3.3
	Moderate	21	17	81	1	4.8	7	33.3	0	0
	Weak	13	12	92.3	0	0	5	38.5	2	15.4
	None	4	4	100	0	0	2	50	0	0

	Total	68	63	92.6	4	5.9	32	47.1	3	4.4
